# Comparing supermarket loyalty card data with traditional diet survey data for understanding how protein is purchased and consumed in older adults for the UK, 2014–16

**DOI:** 10.1186/s12937-020-00602-3

**Published:** 2020-08-13

**Authors:** Mark A. Green, Anthony W. Watson, Jeffrey M. Brunstrom, Bernard M. Corfe, Alexandra M. Johnstone, Elizabeth A. Williams, Emma Stevenson

**Affiliations:** 1 Geographic Data Science Lab, School of Environmental Sciences, University of Liverpool, Liverpool, UK; 2 Human Nutrition Research Centre, Institute of Cellular Medicine, Newcastle University, Newcastle Upon-Tyne, UK; 3 School of Psychological Science, University of Bristol, Bristol, UK; 4 Department of Oncology & Metabolism, University of Sheffield, Sheffield, UK; 5 The Rowett Institute, University of Aberdeen, Aberdeen, UK

**Keywords:** Protein, Big data, Supermarket loyalty cards, Diet surveys, Population

## Abstract

**Background:**

Our ability to understand population-level dietary intake patterns is dependent on having access to high quality data. Diet surveys are common diet assessment methods, but can be limited by bias associated with under-reporting. Food purchases tracked using supermarket loyalty card records may supplement traditional surveys, however they are rarely available to academics and policy makers. The aim of our study is to explore population level patterns of protein purchasing and consumption in ageing adults (40 years onwards).

**Methods:**

We used diet survey data from the National Diet and Nutrition Survey (2014–16) on food consumption, and loyalty card records on food purchases from a major high street supermarket retailer (2016–17) covering the UK. We computed the percentage of total energy derived from protein, protein intake per kg of body mass, and percentage of protein acquired by food type.

**Results:**

We found that protein consumption (as the percentage of total energy purchased) increased between ages 40–65 years, and declined thereafter. In comparison, protein purchased in supermarkets was roughly 2–2.5 percentage points lower at each year of age. The proportion of adults meeting recommended levels of protein was lowest in age groups 55–69 and 70+. The time of protein consumption was skewed towards evening meals, with low intakes during breakfast or between main meals. Meat, fish and poultry dominated as sources of protein purchased and consumed, although adults also acquired a large share of their protein from dairy and bread, with little from plant protein.

**Conclusions:**

Our study provides novel insights into how protein is purchased and consumed by ageing adults in the UK. Supermarket loyalty card data can reveal patterns of protein purchasing that when combined with traditional sources of dietary intake may enhance our understanding of dietary behaviours.

## Introduction

Healthy ageing is a major priority of the UK government [1, 12] and has received much attention due to an ageing population structure. An ageing population has the potential to place considerable strain on health systems if individuals develop multiple chronic conditions. One important aspect of healthy ageing is sarcopenia, which is the decline in muscle mass and strength associated with ageing [6, 14, 24]. Muscle mass not only influences physical functioning, but is also associated with improved survival outcomes in individuals with chronic illnesses [28].

While the determinants of sarcopenia are numerous and complex, diet is one of the major factors influencing the onset and rate of decline in muscle mass. More specifically, dietary protein intake is important in the maintenance and growth of muscle mass and strength especially when paired with weight-bearing exercises [16, 21]. Maintaining adequate protein intake in ageing adults might help to minimise the onset and impact of sarcopenia [17]. Higher absolute protein consumption in ageing adults has also been demonstrated to offer wider health-related benefits, including improved functional ability [18] and a reduced risk of frailty [5].

Understanding how adults consume protein as they age represents an important step for informing policy recommendations [26]. However, few studies have provided a comprehensive examination of population-level protein intake. Many studies focus on smaller scale assessments that are not always generalizable to the wider population and therefore limit their utility for national level decision making. Furthermore, the few studies that have explored population trends have been US centric that may be less applicable to other countries (e.g. varying demographics or social contexts). For instance, it has been shown that in the US protein intake declines with age [15]. Few studies have extended this understanding to see how protein intake varies by other important factors such as sex, socioeconomic deprivation, body mass index or dietary source of protein. Recently published data from the UK have indicated the inadequacy of protein intake in UK older adults, aged 65–89 years, with fewer than 50% of participants meeting current UK recommendations for protein [23]. Targeted intervention to improve the timing of protein intake, for example in the morning, to improve muscle protein synthesis requires food-based solutions that are suitable for older lifestages. This is challenging since consumers eat food and beverages, and not protein per se; protein is a nutrient term that is not well understood by older consumers [4]. Collaboration in the food system between food producers and retailers with academics can help bridge and inform these solutions. New data solutions using big data can provide valuable insight to inform future food solutions.

Our ability to understand and interpret population-level protein behaviours is dependent on having access to high quality data. Diet surveys are common methods for understanding nutrition patterns. Many of these surveys involve self-reported dietary assessment methods that can be biased by under-reporting [3, 20]. We therefore require a range of data sources and types to augment analyses of traditional data sources that might help to minimise their limitations. Furthermore, collecting large dietary survey data is complex, costly and places burdens on participants (e.g. food diaries recorded over multiple days), which may limit their scope and scale for understanding population patterns. New forms of (big) data being generated incidentally by commercial firms may offer the opportunity to supplement traditional data collection approaches, diversify our evidence sources and reduce burdens on participants [8, 27].

With the majority of grocery sales conducted in supermarkets and 65% of people in the UK owning at least one supermarket loyalty card [31], loyalty cards routinely collect information on the food purchasing habits of millions of individuals. The greater scope these big data offer can be powerful for studying diverse contexts or capturing under-representative populations often missed in traditional data sources. For example, the National Diet and Nutrition Survey (NDNS) recruits roughly a thousand individuals per year. The NDNS’s relatively low sample size makes it difficult to focus in detail when analysing subgroups (e.g. older adults) or smaller marginalised populations (e.g. by ethnic group) as they will represent very few participants in the survey (especially if further subgroup analyses are warranted). Recent advances by supermarkets to link purchases to nutritional databases have improved the potential value of purchasing data for assessing dietary patterns and supplementing traditional data sources. This is important as food purchased is not always recorded as consumed during self-reported diet surveys [3, 20], as well as having implications for how policy makers should be targeting the availability of products in shops or behavioural determinants relating to consumption.

Few researchers have access to such data, which has resulted in very few studies exploring the applications of these data [27]. To our knowledge, there is only one study which is based on publicly available grocery sales data for London [2]. These data are limited because: (i) they cover one unrepresentative region meaning no national level recommendations can be made (important as this is the scale nutritional policy is often made at), (ii) data have been aggregated to small areas (e.g. taking mean values on nutritional data) that do not allow conclusions to be drawn on how individuals behave or how purchasing varies by age group. We are not aware of any study that has investigated protein purchasing behaviours in these data type. Hence, determining the usefulness of supplementing traditional dietary assessment methods with loyalty card records for studying dietary behaviours is needed.

The aim of our study is to explore population level patterns of protein purchasing and consumption in ageing adults (40 years onwards). We focus on three main areas: (1) the amount of protein purchased, (2) the timing of protein consumption, and (3) the sources of protein in specific foods. We elected to focus on adults 40 years onwards because evidence suggests that the ageing and gradual decline in muscle mass begins from 40 years onwards [13, 21]. Interventions designed to promote healthy ageing need to target different life course stages, particularly as earlier dietary changes may be more effective at mitigating the longer-term drivers of sarcopenia.

## Methods

### Dietary consumption data

The National Diet and Nutrition Survey (NDNS) is a UK representative rolling cross-sectional diet survey. Participants complete a 4-day diary recording all foods and drinks they consume. Participants also complete a second survey on their personal, social and health characteristics. While the survey is collected annually, we combined survey waves 7 (2014/15) and 8 (2015/16) to improve the stability of estimates due to the small sample size.

We selected all adults aged 40 years and above from the survey sample (*n* = 624). Estimates of nutritional content were provided in the survey dataset, allowing us to examine the proportion of total energy intake from protein foods (both measured in kcal). We also calculated the ratio of protein consumed (g) per kg body weight of individuals. Body weight was self-reported by participants. Sample weights to account for non-response sampling bias provided in the NDNS were used when calculating estimates to help maintain the representative nature of the survey. We measured socioeconomic deprivation using the 2015 English Index of Multiple Deprivation (IMD), a multidimensional index of neighbourhood deprivation used by the UK government [22]. Our sub-analyses by socioeconomic status (SES) are for England to match the supermarket data, as the loyalty card records were supplied with linked deprivation data only for English records by the retailer.

### Food purchasing data

Loyalty card data were supplied by one of the largest UK high street supermarkets. The retailer has shops throughout the UK (including both large supermarkets and smaller convivence stores) and sells a diverse range of goods including fresh and long-life foods. Loyalty card schemes are used by retailers to build brand loyalty by providing incentives (e.g., price coupons) based on shopping experiences [8]. They also enable retailers to understand demand in their stores, and to offer targeted incentives to individual customers [27]. When customers purchase products and present their loyalty card, the purchase is logged to a central system and recorded against the loyalty card holder. Customers must also provide additional details such as date of birth, sex, and residential address upon applying for a loyalty card.

Data were provided covering the financial year 13th March 2016 to 11st March 2017. We were supplied a fully anonymised data set that included the annual total of purchases made by all customers with a loyalty card who were residents in the UK. Variables included the total energy of items purchased, total protein purchased from all products, and the equivalent of these two measures split by product category (no other nutrient information was supplied). Product category was defined by the retailer and was aggregated to broad categories (Additional file 1). The categories were not directly comparable to the NDNS data. Total energy and protein content of individual food items is provided by product suppliers to the retailer and linked to the purchase data to allow the calculation of nutrient composition of purchases. Nutritional information is provided per 100 g and per pack allowing for the calculation for weighed and packed items.

Age, sex and IMD quintile (same measure as in the NDNS) of loyalty card holder’s details were also supplied (defined at 13th March 2016). We were only provided IMD for England by the supermarket and so have restricted these analyses to England only for consistency (these analyses are presented in the Appendices only).

### Statistical analyses

Descriptive statistics were generated to examine intake patterns. We also provide smoothed estimates to reduce the impact of noisy estimates. Smoothing was undertaken using LOESS regression. All analyses were conducted using R and all analytical code can be located on https://github.com/markagreen/protein_for_life.

## Results

### Amount of protein

**Fig. 1:**
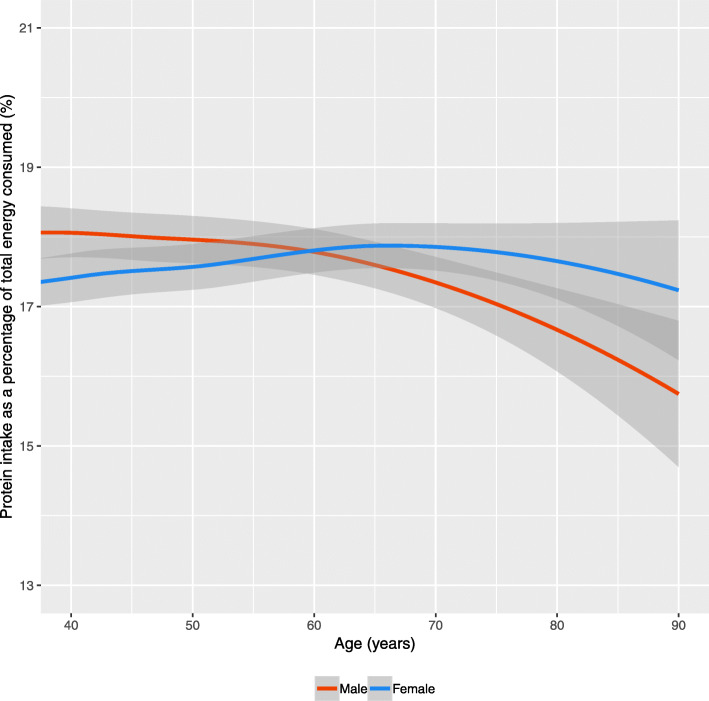
Protein intake as a percentage of total energy consumed by age and sex for the UK (Data: National Diet and Nutrition Survey, 2014–16). Note: Smoothing was completed using a LOESS regression with 95% Confidence Intervals

For both males and females, the protein intake as a percentage of total energy consumed remained flat between ages 40 and 60 years (Fig. 1). Sex-based trends then begin to diverge from 60 years onwards, with protein contributing less total energy for men (the decline is two percentage points by 90 years). For females, the trend remains fairly consistent over the period, with a modest decline from 80 years onwards (~ 0.5 percentage points). These relative trends occur within the context of declining absolute levels of protein intake and total energy from food with increasing age group (Additional file 1). Stratification by IMD suggests small and inconsistent differences by SES (Additional file 1).

**Fig. 2:**
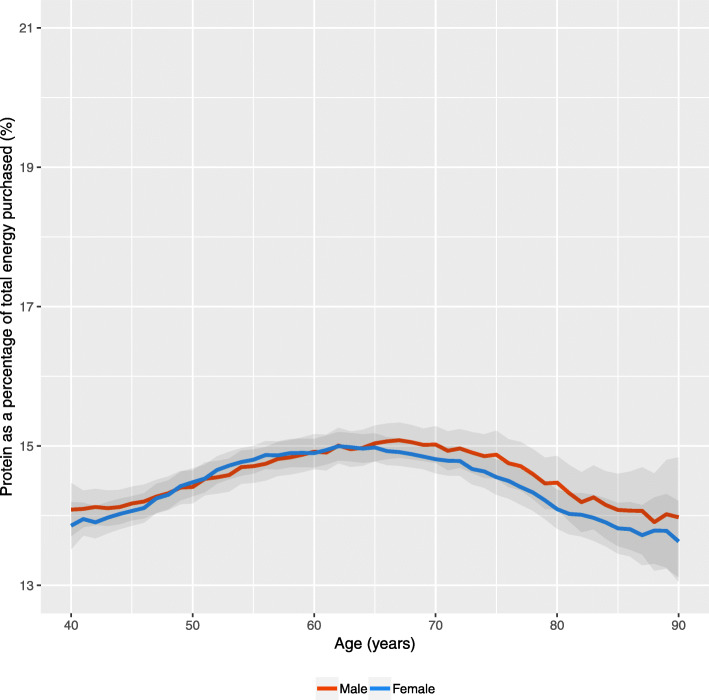
Protein as a percentage of total energy purchased by age and sex for the UK (Data: High Street Supermarket, 2016–17). Note: Data are not smoothed due to the larger sample size and include 95% Confidence Intervals

Figure 2 presents the same measure for the high street retailer data. Notably, the percentage of energy purchased that was protein is far lower than the estimates from the consumption data (roughly 2–2.5 percentage points lower at each individual year of age than the smoothed estimates in Fig. 1). The shape of the distribution by age is fairly similar, increasing between 40 and 60 years, and declining thereafter (although not as sharp as in Fig. 1 with ~ 1 percentage point variation). Differences by sex were smaller, and while males purchased a larger share after age 60 (contrasting the consumption data) the extent of the difference is small and insignificant. Stratifying the analysis by IMD quintile resulted in minimal differences (Additional file 1).

The consumption (NDNS) data allowed us to examine the proportion of individuals who were meeting protein intake guidelines. Table 1 presents varying levels of protein intake (accounting for body mass) that could be used as guidelines; we consider 0.75 g per kg body mass which is the recommended intake in the UK [9], 0.8 g/kg which is the international recommended dietary allowance [30], and then subsequent 0.1 g/kg increases up to 1.2 g/kg in line with European Society for Clinical Nutrition and Metabolism recommendations [11]. We combined individuals into broad age groups representing different life-stages. Few individuals met the higher recommended values. The proportion meeting each level is lowest in the oldest age groups. Males were more likely to meet each level of protein intake.

**Table 1:** Percentage of adults in the UK meeting different levels of protein intake per body weight (Data: National Diet and Nutrition Survey, 2014–16)

Sex	Age group (years)	Percentage (%) meeting recommended total protein intake (g) per kg body mass					
		0.75	0.8	0.9	1.0	1.1	1.2
Male	40–54	82.1	76.2	62.8	47.9	35.8	25.1
	55–69	76.6	68.7	51.1	33.8	21.9	09.8
	70+	78.1	65.3	53.1	40.7	25.6	14.7
Female	40–54	71.1	68.5	54.3	39.5	28.0	20.3
	55–69	71.5	64.8	48.0	38.0	29.4	12.9
	70+	64.3	59.3	44.8	30.5	19.1	15.8

### Timing of protein

**Fig. 3:**
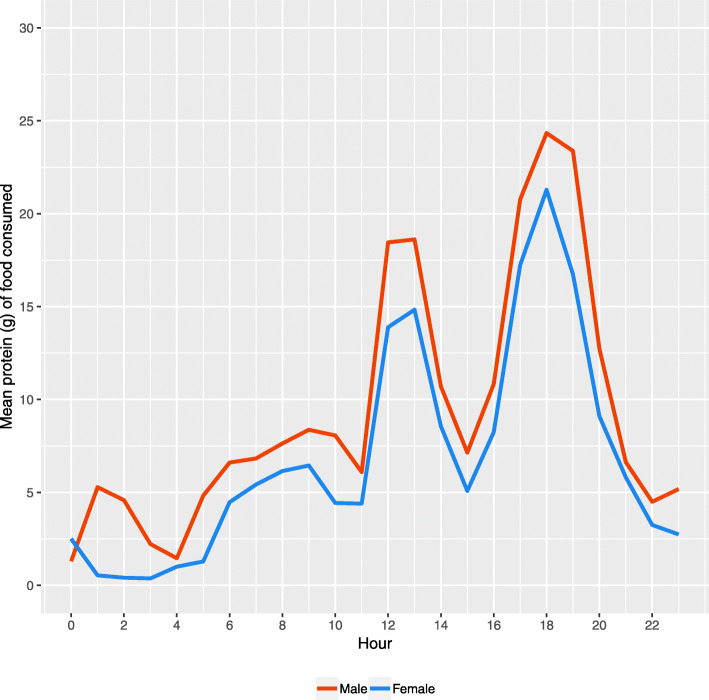
Mean consumption of protein (g) by hour of day and sex for the UK (Data: National Diet and Nutrition Survey, 2014–16)

The NDNS required participants to record the time of food consumption, allowing us to examine the temporal variability in protein intake. Figure 3 presents mean protein intake by hour of day. Protein consumption was highest for the period associated with evening meals (5-7 pm). This was accompanied by a second large spike in consumption during lunchtime (12-1 pm). The morning/breakfast period also represented a period of higher protein consumption, although not as high as the other two periods. Protein intake in-between these three time periods was low (< 10 g). There were minimal differences by sex. We stratified the analysis by age group (40–54, 55–70, 70+), however the patterns remained the same (Additional file 1).

### Sources of protein

Finally, we examined where individuals were sourcing their protein from. Our two data sources were not directly comparable. Table 2 presents the common sources of protein consumed in the NDNS (stratified by age group and sex). Food sources were dominated by meat, fish and poultry, as well as dairy and bread products. Chicken and turkey dishes were the major source for both males and females however, intake declined across age groups. Red meats and dairy products accounted for a larger share in the older age groups (this pattern was stronger for males). There was low plant-based protein intake.

**Table 2:** Percentage of protein acquired from the most common food sources by sex and age group (years) for the UK (Data: National Diet and Nutrition Survey, 2014–16). Note: Food groups are presented in decreasing order of percentage by sex and 40–54 years age band

Food	Males			Food	Females		
	40–54	55–69	70+		40–54	55–69	70+
Chicken and turkey dishes	14.6	11.5	8.0	Chicken and turkey dishes	15.2	11.0	10.5
White bread	6.3	7.1	5.5	Beef and veal dishes	6.1	6.3	6.9
Pasta, rice and other cereals	5.9	4.0	4.0	Pasta, rice and other cereals	6.0	5.2	3.3
Beef and veal dishes	5.8	7.9	8.1	Semi skimmed milk	5.4	6.1	8.6
Semi skimmed milk	4.9	5.2	7.5	White bread	5.3	4.3	5.9
Vegetables not raw	4.7	4.6	3.7	Vegetables not raw	5.1	5.2	4.1
Cheese	4.4	4.3	5.3	Cheese	4.7	5.2	5.7
Bacon and ham	4.4	5.0	5.9	Bacon and ham	4.6	3.4	3.6
Eggs and egg dishes	3.9	4.0	4.3	Eggs and egg dishes	4.5	4.1	4.3
Other white fish and shellfish dishes	3.1	3.2	2.6	Other white fish and shellfish dishes	3.2	3.6	2.9
Brown granary and wheatgerm bread	2.3	2.8	2.5	Pork and dishes	2.5	2.8	2.3
Oily fish	2.3	2.6	3.9	Brown granary and wheatgerm bread	2.2	2.6	2.0
Burgers and kebabs	2.1	0.8	0.2	Miscellaneous (see Additional file 1)	2.2	1.9	1.8
Sausages	2.1	2.1	2.3	Oily fish	1.9	3.9	3.9
Pork and dishes	2.1	2.9	3.0	Sausages	1.7	1.6	1.2
Miscellaneous	2.0	2.0	2.1	Other potatoes, potato salads & dishes	1.6	1.7	2.5
White fish coated or fried	1.9	1.9	1.7	Nuts and seeds	1.4	1.6	0.6
Lamb and dishes	1.9	1.5	0.9	Fruit	1.4	1.9	1.6
Beer, lager, cider & perry	1.8	1.8	0.8	Biscuits	1.4	1.4	1.6
Chips, roast potatoes and potato products	1.6	1.6	1.3	Lamb and dishes	1.4	1.6	2.5

**Fig. 4:**
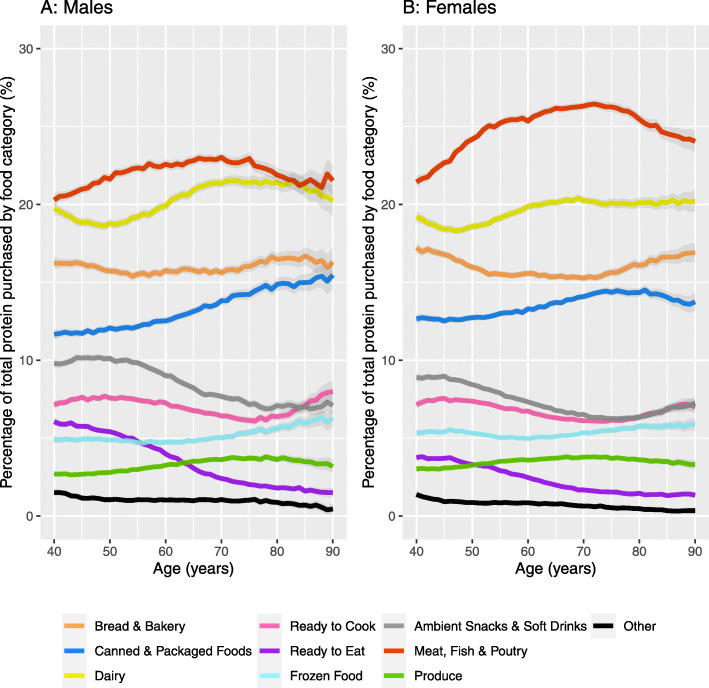
Percentage of total protein purchased across food group by age for males (**a**) and females (**b**) in the UK (Data: High Street Supermarket, 2016–17). Note: Data are not smoothed due to the larger sample size and include 95% Confidence Intervals

Figure 4 shows protein purchasing source by sex. Meat, fish and poultry had the largest share of protein intake, although the proportion was larger for females compared to males. Both males and females displayed similar patterns across age groups with an increasing share of protein purchased due to meat, fish and poultry between 40 and 65 years, and declining thereafter. Dairy, the second largest share, was larger for males (although most pronounced in respondents aged 60 years or older) and almost converges with meat, fish and poultry in the 80+ years male group. Bakery constituted the third largest share with a similar and almost ‘u’ shaped pattern for both males and females. There is rising share of protein from ‘canned and packaged goods’ with age group, with small increases also observed for frozen foods and produce. These patterns are partly explained through declines in perishable categories such as ‘food for later’ and ‘food to go’.

Stratifying the analyses by IMD quintile (Additional file 1) revealed differences in purchasing behaviours by deprivation. Both males and females in the least deprived quintile had a higher share of protein from meat, fish and poultry sources between 40 and 65 years, at which point the values begin to converge to the most deprived quintile of areas. Individuals from the least deprived quintile of areas also had a larger share of protein from dairy from 60 onwards, canned and packaged goods in most age groups, and marginally for food for later goods in ages 40–60. Individuals from the most deprived quintile had a larger share of protein from bakery goods and frozen food.

## Discussion

### Key results

Our study provides a comprehensive exploration of population-level patterns of protein purchased and consumed in older adults in the UK. We find a decline in the percentage of energy derived from protein both consumed and purchased in the oldest age groups, and similarly fewer adults in the two oldest age groups meeting recommended protein intake levels. A higher proportion of protein was reported in the consumption data compared to the purchasing data. Protein purchasing and consumption was largest for meat and poultry products, but was also high for dairy and bread/bakery items as well. There was low purchasing and consumption of plant proteins.

### Interpretation

Our findings reveal a nuanced association between age group and protein behaviours. Protein consumption declined with increasing age group for males, but not for females (Fig. 1). In contrast, declining protein purchasing from 60 years onwards was consistent by sex (Fig. 2). The proportion of individuals meeting protein recommendations declined linearly with age group for females, but not for males (Table 1). Declines in protein behaviours by age group were not consistent across all sources of protein (Table 2 and Fig. 4). Designing interventions around protein and age group may help to elucidate these complexities.

Evidence of lower levels of protein consumption and purchasing in the oldest age group represents an important area for policy makers. Protein intake is associated with the degree of muscle decline in ageing adults [16], and therefore represents an important determinant of sarcopenia. Dietary interventions might be more feasible and less intrusive than strength training in older adults [6]. However, focusing purely on diet ignores the complex reasons for this pattern including lower energy needs in older adults, difficulty in preparing meals, underlying comorbidities, changing preferences, and changes in dentition that impact the ability to consume certain foods [28]. Protein consumption is linked to satiety and therefore any intervention would need to offset potential declines to energy consumption. Furthermore, since sarcopenia is promoted by food choices over many years, to be most effective interventions need to target behavioural change much earlier in the life course [26].

The lower percentage of protein found in the purchasing data compared to the consumption data present novel findings. Few studies have previously compared these data types due to a lack of availability of loyalty card data [27]. It was not clear why the difference in consumption and purchasing data might exist. A variety of reasons include individuals underreporting what they consume (e.g. underreporting consumption of purchased non-protein sources) [3, 20], different sample years or the varying sociodemographic makeup of individuals within each dataset. Our purchasing data was closer than our consumption data to reported values in the US (~ 15%) [6]. They may also reflect broader consumer dynamics, such as individual’s purchasing protein from alternative retail outlets than just the high street supermarket (e.g. local butchers). People purchasing foods may not be buying foods that they will necessarily consume; household composition matters (data may be more accurate for single or dual person households than families). Finally, differences may reflect food wastage patterns where high value meat is prioritised over bread, fruit and vegetables [25].

We find evidence that not all adults meet a range of protein recommendations, with patterns declining with age group as well. While our findings contrast to evidence from the UK Biobank [7], UK Biobank is not a representative survey and our findings match results from other contexts [15, 23]. There are currently no UK guidelines on recommended protein intake that take into account ageing [21]. Improving the clarity of messaging around protein might help encourage adequate consumption of protein in older adults. However, defining the expected level of protein is difficult. While the international recommended dietary allowance of 0.8 g/kg is set at two standard deviations above the minimum amount of protein to maintain body protein and loss of nitrogen [28], this figure does not necessarily represent optimal intake or reflect evidence that older adults would benefit from greater amounts of protein intake (not solely for muscle maintenance, but also broader health benefits) [6, 11, 16, 19, 29]. Focusing on the more stringent protein guidelines we examined might be more beneficial, particularly given that a minority of individuals currently meet such recommendations.

Timing of protein was skewed towards evening meal, and to a lesser extent lunch meals. This finding follows previous research [10]. On average, individuals were not meeting the recommended 25-30 g of protein required to maintain muscle mass and function at either of these periods [21, 24]. Protein intake during breakfast was particularly low, suggesting an opportunity to target this meal in interventions [10]. The low levels of protein in-between main meal times also present opportunities for interventions. Smaller but more regular protein snacks throughout the day may help individuals increase their protein consumption [21]. While many protein-rich snacks exist, they tend to be focused towards high performance and athletes, which limits their general appeal [26].

Finally, our study provides novel insight into how supermarket loyalty card data may aid our understanding of protein-related behaviours. There has been a lot of excitement about the promise of big data, however there have been few applications in nutrition-related studies so far [27]. Our study reveals nuances in purchasing behaviours that change across age groups, which both support and extend the observations in the NDNS. However, the loyalty card data cannot answer all questions surrounding protein; we were unable to assess whether protein purchased was consumed (or who it was purchased for), when it was consumed or how purchasing related to recommended levels of intake. Purchase patterns are likely to be driven by a main shopper, who may act as a gatekeeper for a household. It is clear that these new forms of data can only supplement traditional data and such data will remain important in answering future research questions.

### Limitations

There are several limitations of our study. A non-disclosure agreement was signed for use of the in-store purchase data and we are restricted in the details that we can report here. We were unable to report key sample information including sample size (which was in the order of millions) or basic demographic characteristics of loyalty card holders (which were not representative of the UK population). Not being able to report the representativeness of a sample can limit the ability to scrutinise the quality of the data, which is important when we are making comparisons across data sets. Loyalty card usage varies by age group and sex introducing bias into estimates as well [31].

Loyalty cards may be shared between individuals (e.g. individuals purchasing for friends and family unable to visit supermarkets themselves) and within households reducing their applicability for studying individual-level patterns in purchasing behaviours. This restricts the ability to draw comparisons to the NDNS data and may limit our conclusions. It represents a broader concern with the applications of supermarket loyalty card data and how they might supplement other forms of data [20].

The focus of our paper on protein restricts the wider conclusions we can draw on the use of supermarket loyalty card records as a dietary assessment tool. Although this was not the aim of our study, validating how purchasing patterns for a greater range of macro- and micro-nutrients relate to consumption patterns is needed to evaluate the usefulness of supermarket loyalty card data in diet-related research. Future studies should also consider how patterns vary to other dietary assessment methods and surveys, link loyalty cards to individual-level surveys to allow for direct comparisons, and collect data from multiple loyalty cards to cover all potential transactions.

Our analyses were mainly descriptive and cross-sectional. We are limited in our ability to draw associations between dietary behaviours and age. There are few studies that have examined longitudinal associations of protein intake [21]. Extending our analyses across the life course might help to shed light on reasons why protein consumption changes across age groups. Longitudinal and repeated cross-sectional data would also help to examine trends in dietary behaviours and the context to our data. Examining the existence of cohort effects is also important to determine how protein consumption and purchasing changes across age groups.

## Conclusions

Our study presents a novel comparison of protein behaviours among ageing adults regarding purchasing and consumption of protein. It is one of the first studies to utilise loyalty card supermarket data to investigate dietary behaviours, as well as providing a comprehensive exploration of population-level protein behaviours. Our results have important implications for promoting healthy ageing through protein related dietary behaviours.

## Supplementary information

**Additional file 1.**

## Data Availability

The National Diet and Nutrition Survey datasets supporting the conclusions of this article are available in the UK Data Archive repository, 10.5255/UKDA-SN-6533-15]. We were not allowed to share the supermarket loyalty card records as per our Non-Disclosure Agreement. All R analytical scripts are located on https://github.com/markagreen/protein_for_life.
