# Supplementary material for: Comparing supermarket loyalty card data with traditional diet survey data for understanding how protein is purchased and consumed in older adults for the UK, 2014–16
Source: Nutr J. 2020 Aug 13;19:83. doi: 10.1186/s12937-020-00602-3 (PMC7427066; doi:10.1186/s12937-020-00602-3)
Supplement: Supplementary file 1 — Additional file 1. [file 12937_2020_602_MOESM1_ESM.pdf]

Supplementary Appendices

For the paper:

Comparing supermarket loyalty card data with traditional survey data for understanding how protein is purchased and consumed in older adults for the UK, 2014-16

Mark A Green[1], Anthony W Watson[2], Jeffrey M Brunstrom[3], Bernard M Corfe[4], Alexandra M Johnstone[5], Elizabeth A Williams[4], Emma Stevenson[2]

[1] Geographic Data Science Lab, School of Environmental Sciences, University of Liverpool, Liverpool, UK
[2] Human Nutrition Research Centre, Institute of Cellular Medicine, Newcastle University, Newcastle Upon-Tyne, UK
[3] School of Psychological Science, University of Bristol, Bristol, UK
[4] Department of Oncology, University of Sheffield, Sheffield, Sheffield, UK
[5] The Rowett Institute, University of Aberdeen, Aberdeen, UK

Contents:

Page	Appendix
2	A - Definitions
3	B - Amount of protein and energy from food (absolute values)
4	C - Amount of protein by deprivation quintile
6	D - Timing of protein by age band
8	E - Source of protein by deprivation quintile – males
11	F - Source of protein by deprivation quintile - females

Appendix A - Definitions

We are not allowed to report the exact definitions of the food categories we used due to our data agreement (some of the names have been changed as a result). We offer examples of what they contain below to help their interpretation.

Table A1: Descriptions of high street retailer product categories.

Category	Examples
Baby & Beauty	Baby food, toothpaste, toiletries
Bakery	Bread, pastries, cakes
Beer, wine & spirits	Alcoholic beverages
Canned & Packaged Foods	Soup, canned vegetables, pasta
Customer Ordering	On-line orders (not covered elsewhere)
Dairy	Milk, yoghurt, cheese
Food for Later	Ready meals
Food to Go	Salads, sandwiches
Food Service	Fresh snacks
Frozen Food	Frozen pizza, chips, desserts, vegetables
Impulse	Chocolate bars, crisps, sweets
Meat, Fish & Poultry	Beef, salmon, chicken
Produce	Vegetables, fruit
World Foods	Afro-Caribbean, Asian, Polish specific items

Where we use Index of Multiple Deprivation (IMD) data, we group individuals by the quintile of their ranks. Quintile 1 represents the most deprived areas, and quintile 5 represents the least deprived areas.

In Table 2, the main food group 'miscellaneous' is reported. The food group includes beverages from dried items (e.g. drinking chocolate, milk shake powder), soup (manufactured or homemade), nutrition powders (e.g. protein shakes, meal replacement drinks) and savoury sauces/gravies.

Appendix B - Amount of protein and energy from food (absolute values)

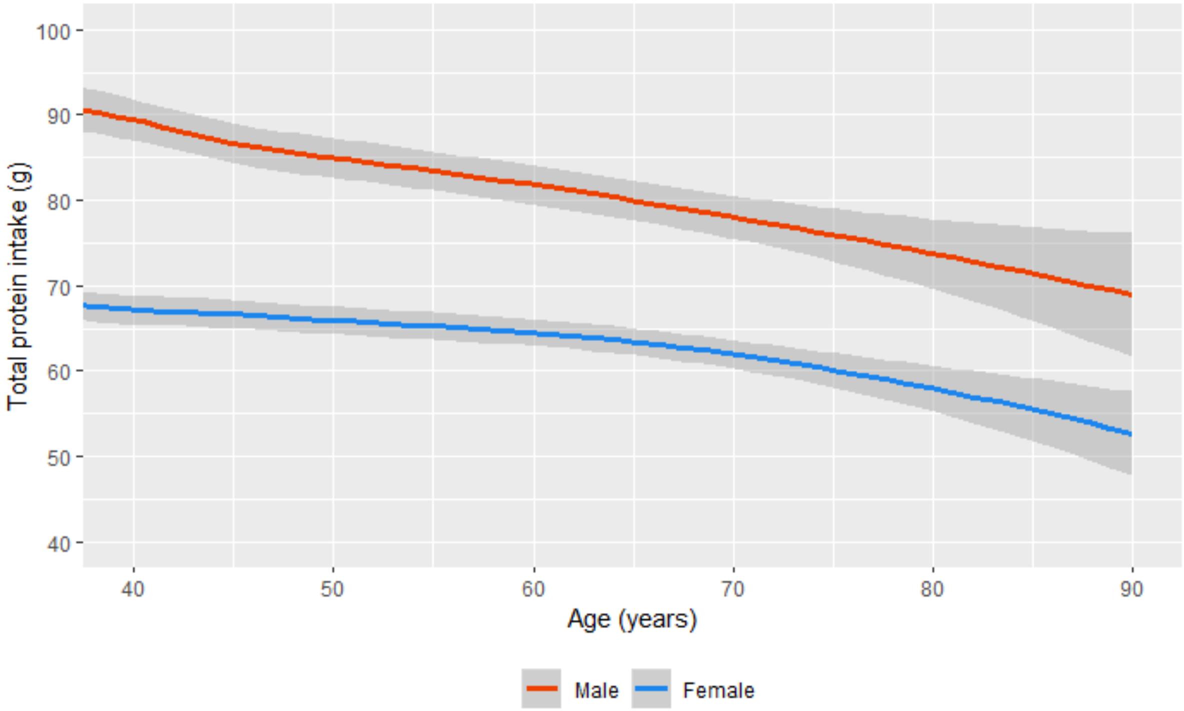

Figure B1: Average daily total protein consumed (g) by age and sex (Data: National Diet and Nutrition Survey, 2012-14).

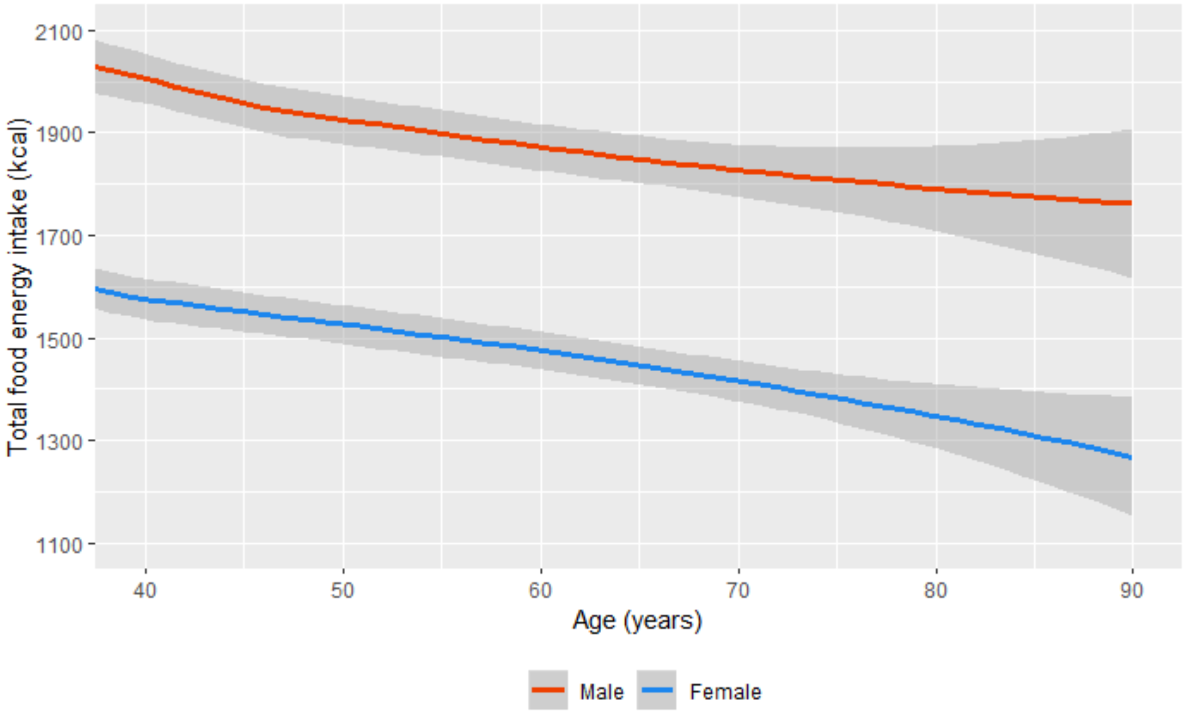

Figure B2: Average total energy consumed from food (kcal) by age and sex (Data: National Diet and Nutrition Survey, 2012-14).

Appendix C - Amount of protein by deprivation quintile

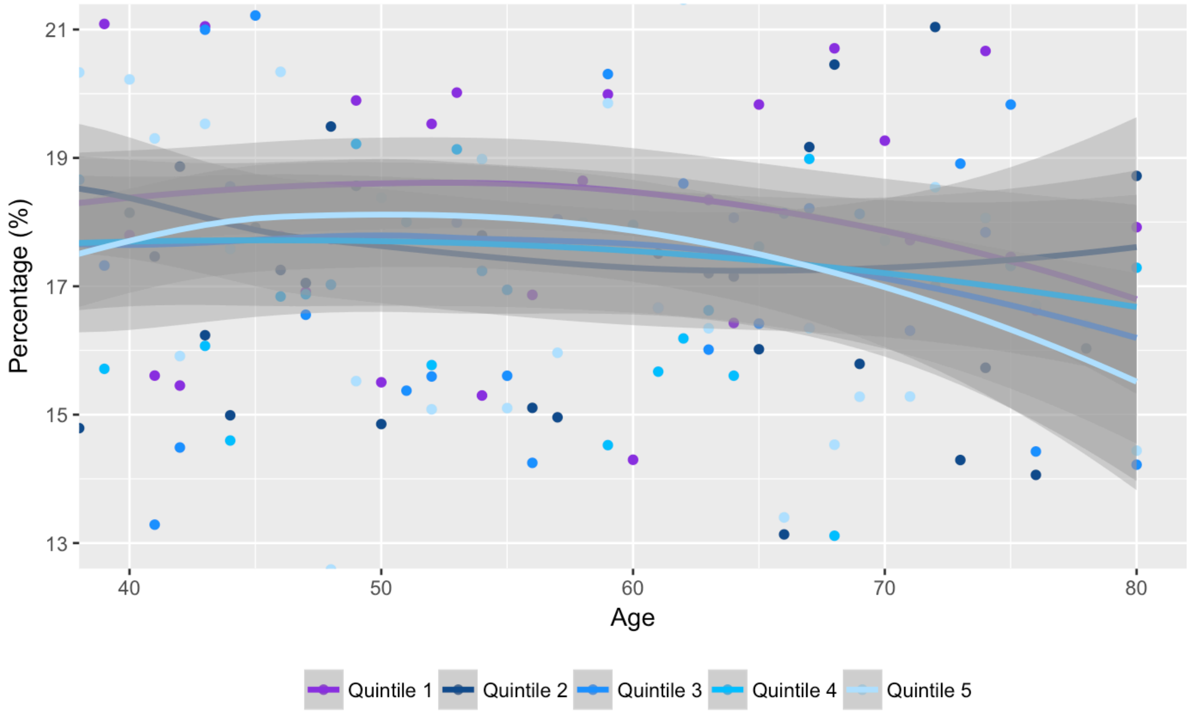

Figure C1: Percentage of total energy consumed that was protein by age and index of multiple deprivation (IMD) quintile for males (Data: National Diet and Nutrition Survey, 2012-14).

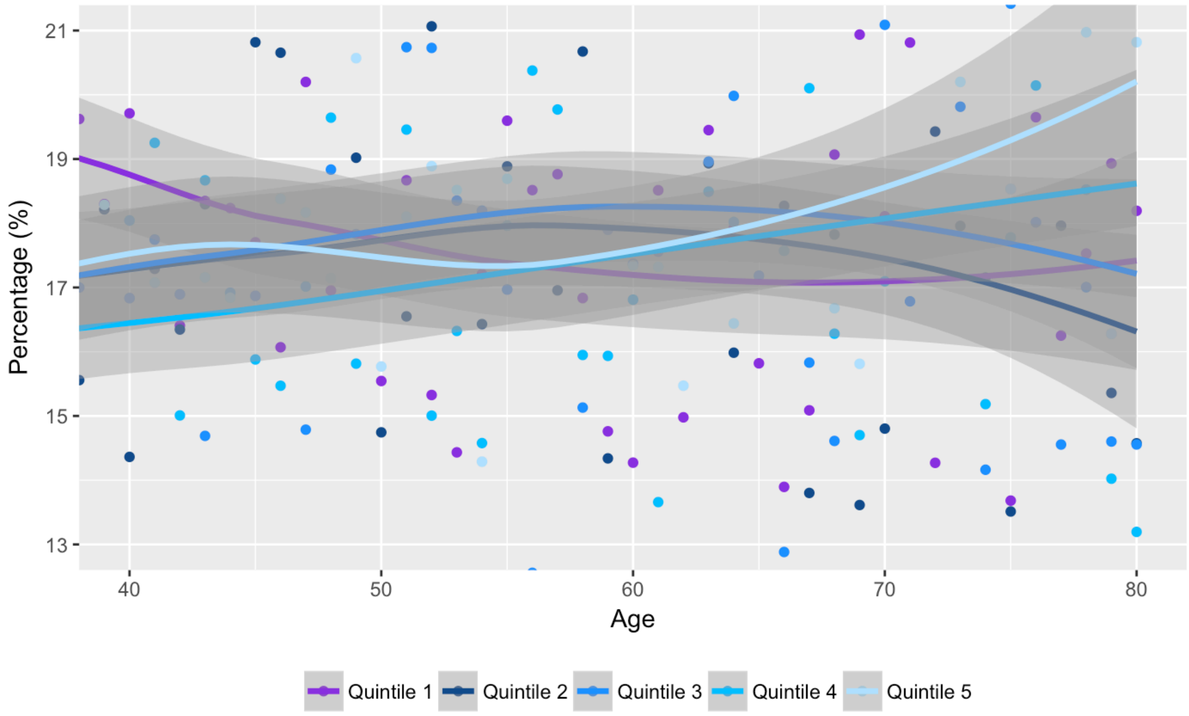

Figure C2: Percentage of total energy consumed that was protein by age and index of multiple deprivation (IMD) quintile for females (Data: National Diet and Nutrition Survey, 2012-14).

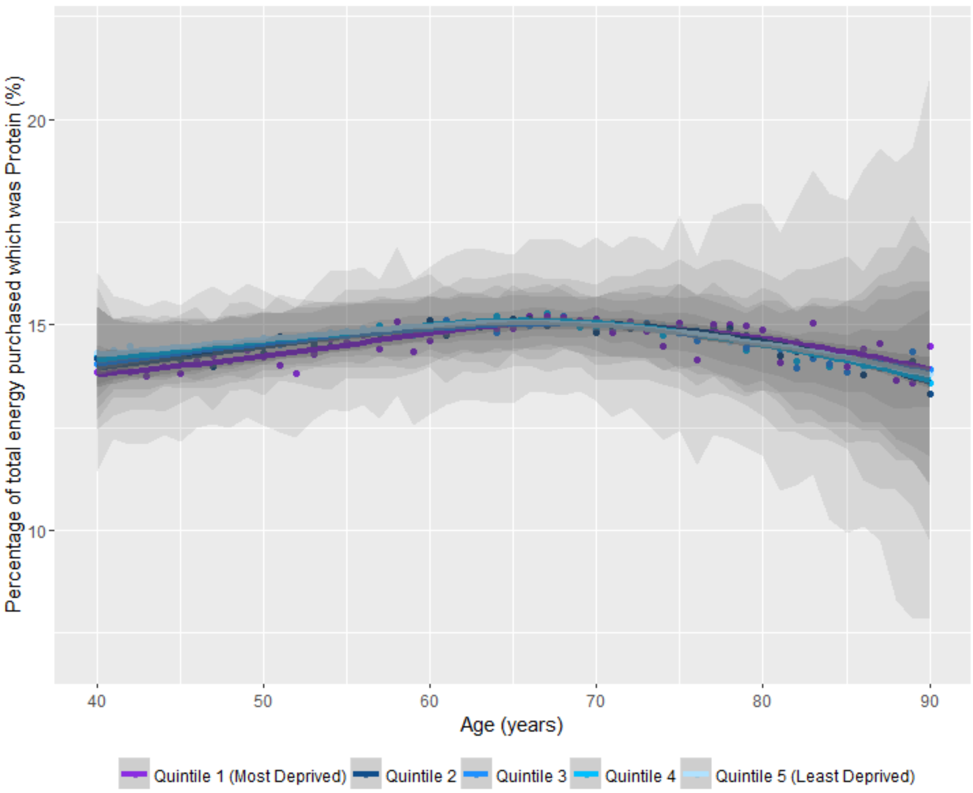

Figure C3: Percentage of total energy purchased that was protein by age and index of multiple deprivation (IMD) quintile for males (Data: High Street Supermarket, 2016-17).

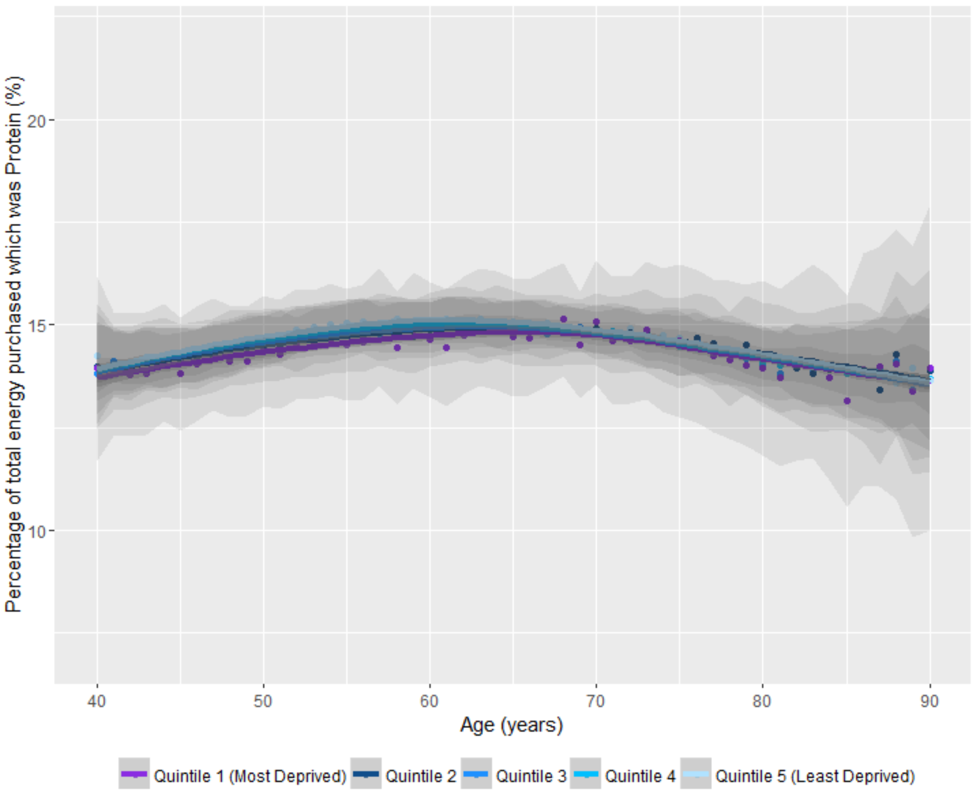

Figure C4: Percentage of total energy purchased that was protein by age and index of multiple deprivation (IMD) quintile for females (Data: High Street Supermarket, 2016-17).

Appendix D - Timing of protein by age band

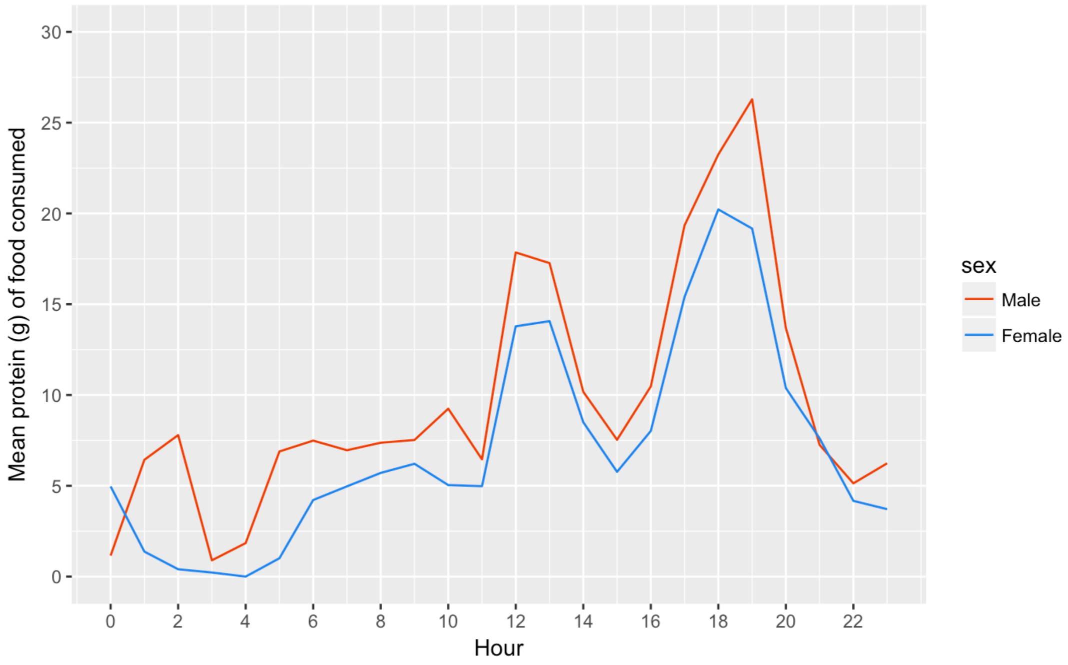

Figure D1: Mean consumption of protein (g) by hour of day of individuals aged 40-54.

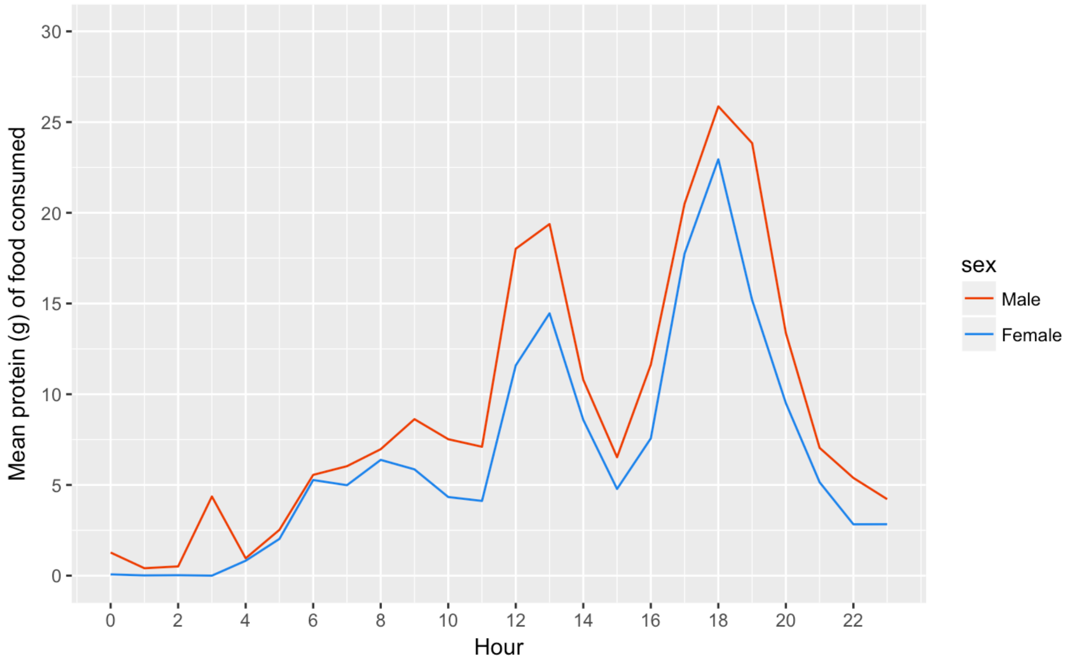

Figure D2: Mean consumption of protein (g) by hour of day of individuals aged 55-69.

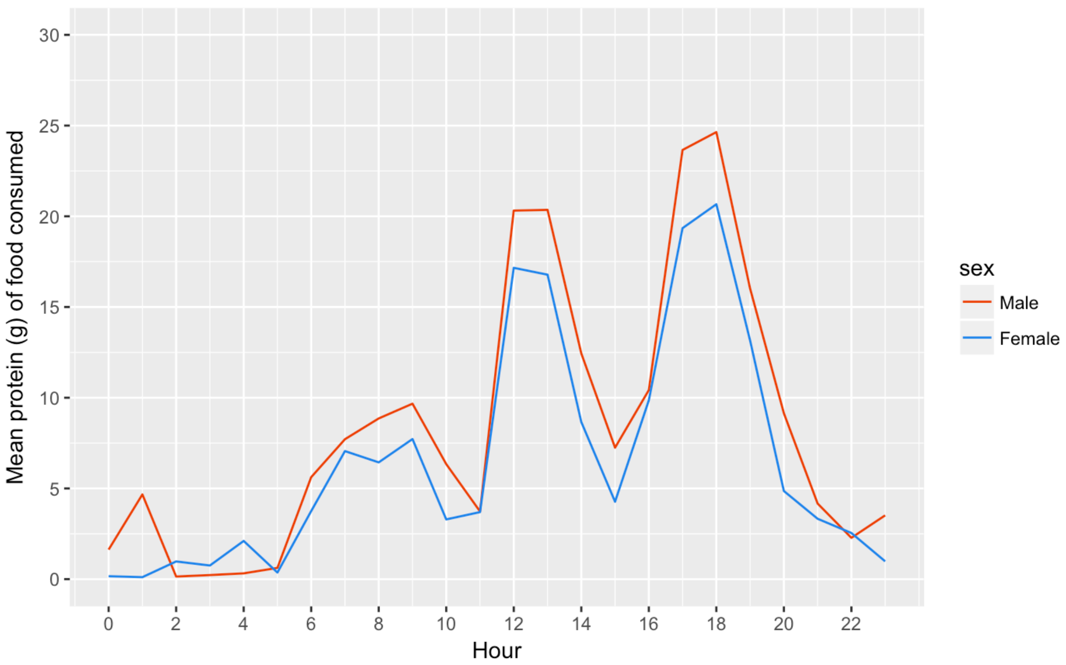

Figure D3: Mean consumption of protein (g) by hour of day of individuals aged 70+.

Appendix E - Source of protein by deprivation quintile - males

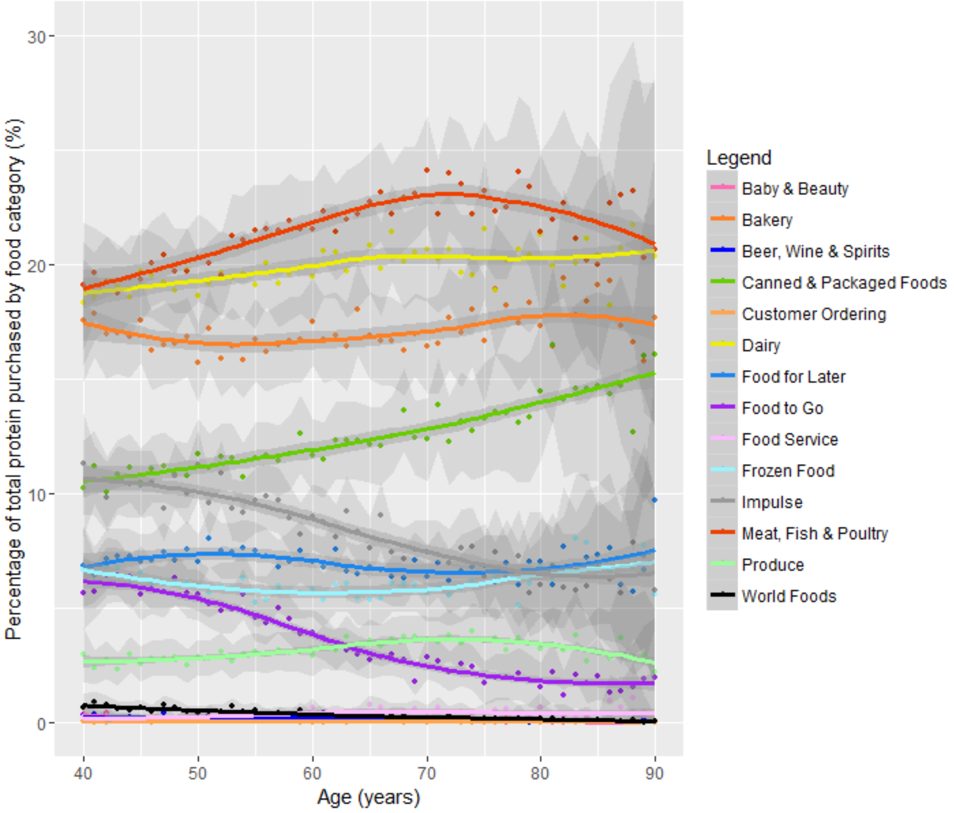

Figure E1: Percentage of protein purchased across food group by age for males in index of multiple deprivation quintile 1 (Data: High Street Supermarket, 2016-17).

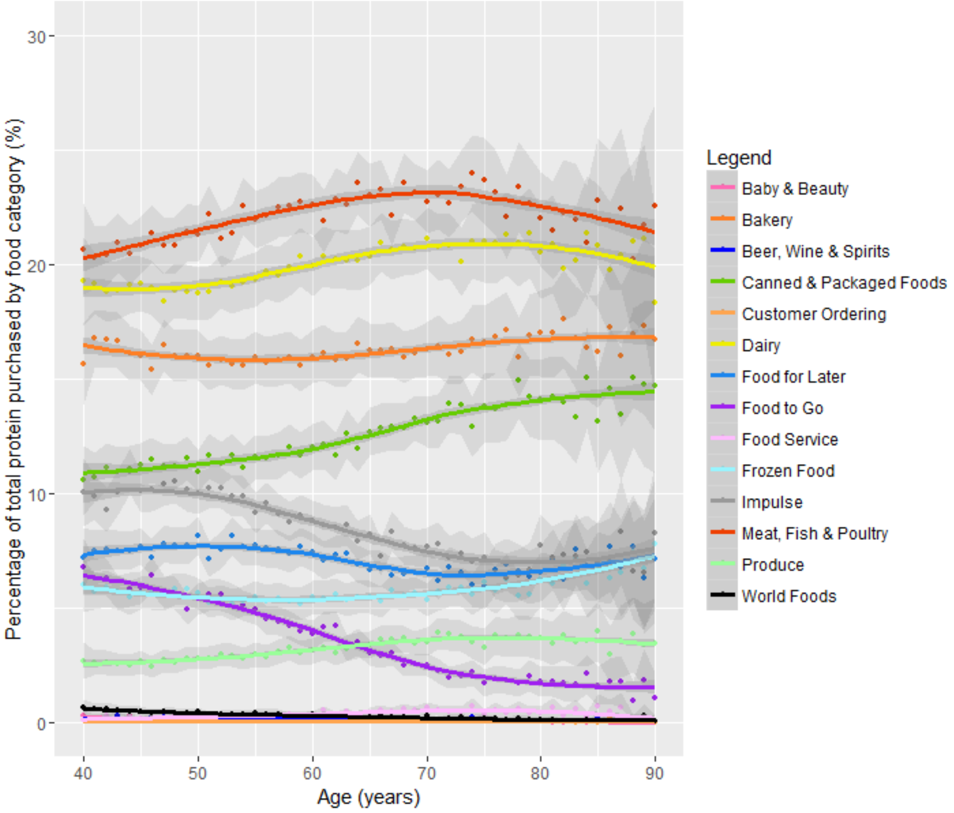

Figure E2: Percentage of protein purchased across food group by age for males in index of multiple deprivation quintile 2 (Data: High Street Supermarket, 2016-17).

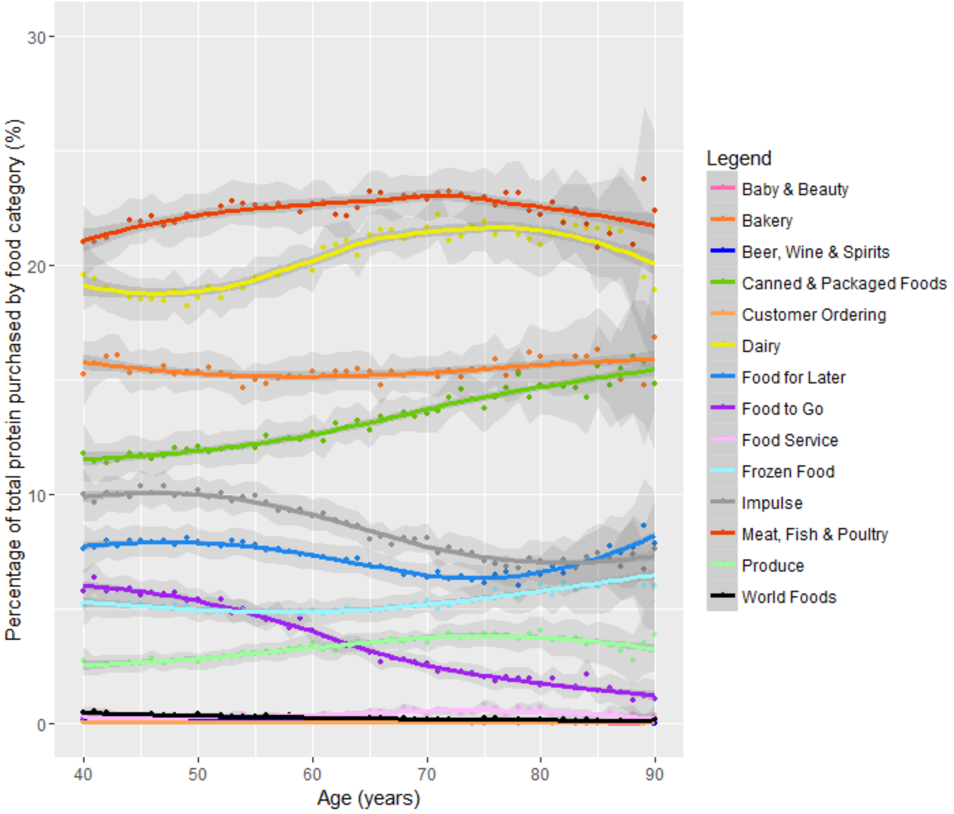

Figure E3: Percentage of protein purchased across food group by age for males in index of multiple deprivation quintile 3 (Data: High Street Supermarket, 2016-17).

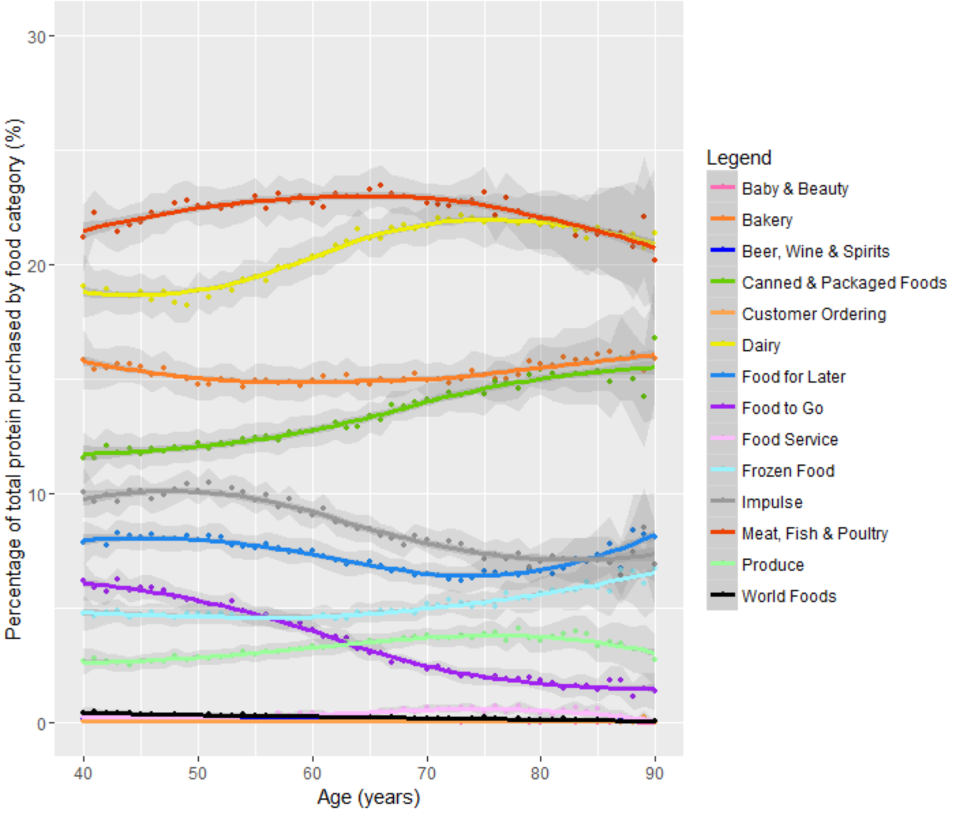

Figure E4: Percentage of protein purchased across food group by age for males in index of multiple deprivation quintile 4.

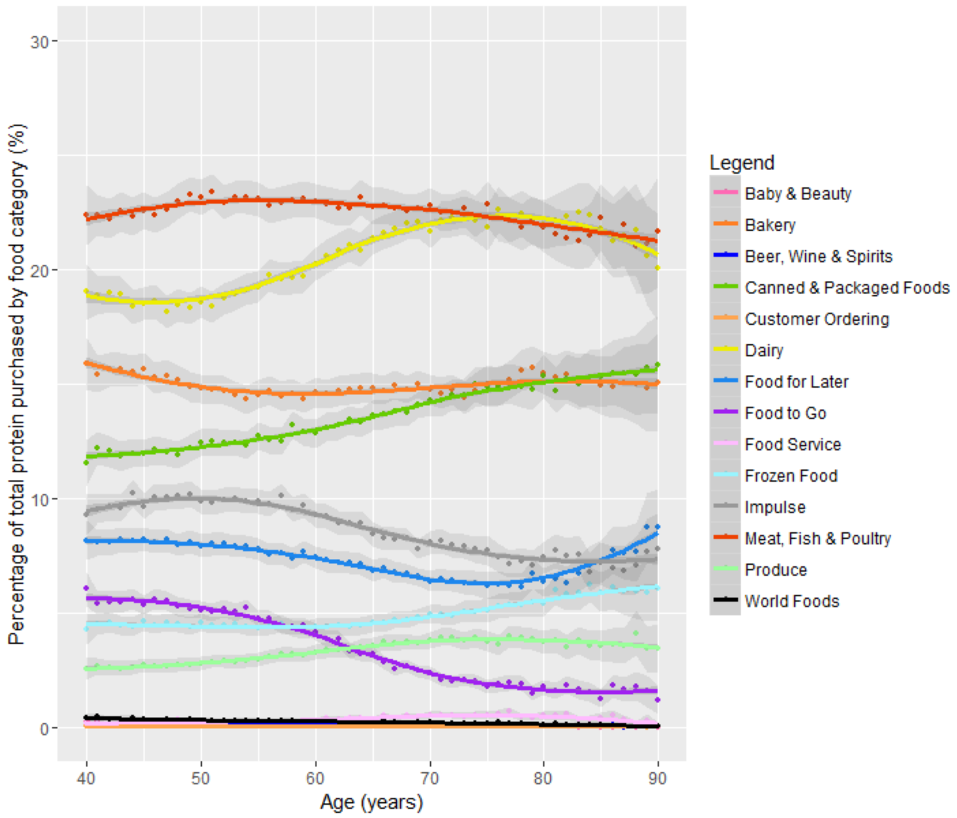

Figure E5: Percentage of protein purchased across food group by age for males in index of multiple deprivation quintile 5 (least deprived quintile).

Appendix F - Source of protein by deprivation quintile - females

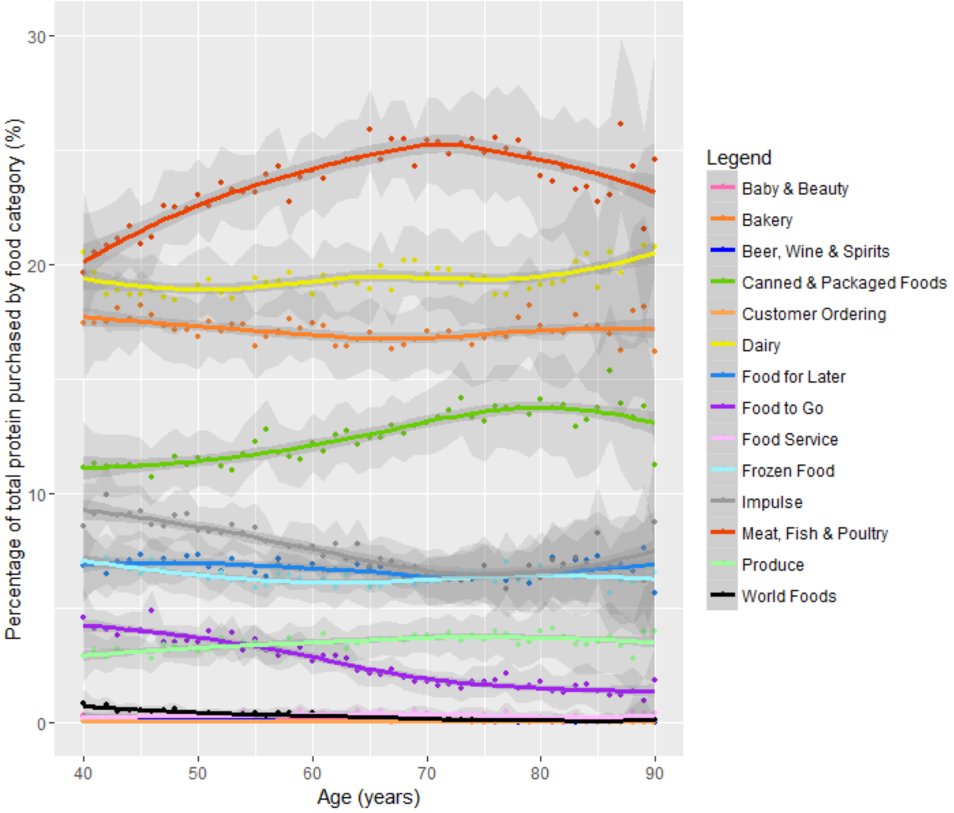

Figure F1: Percentage of protein purchased across food group by age for females in index of multiple deprivation quintile 1 (most deprived quintile).

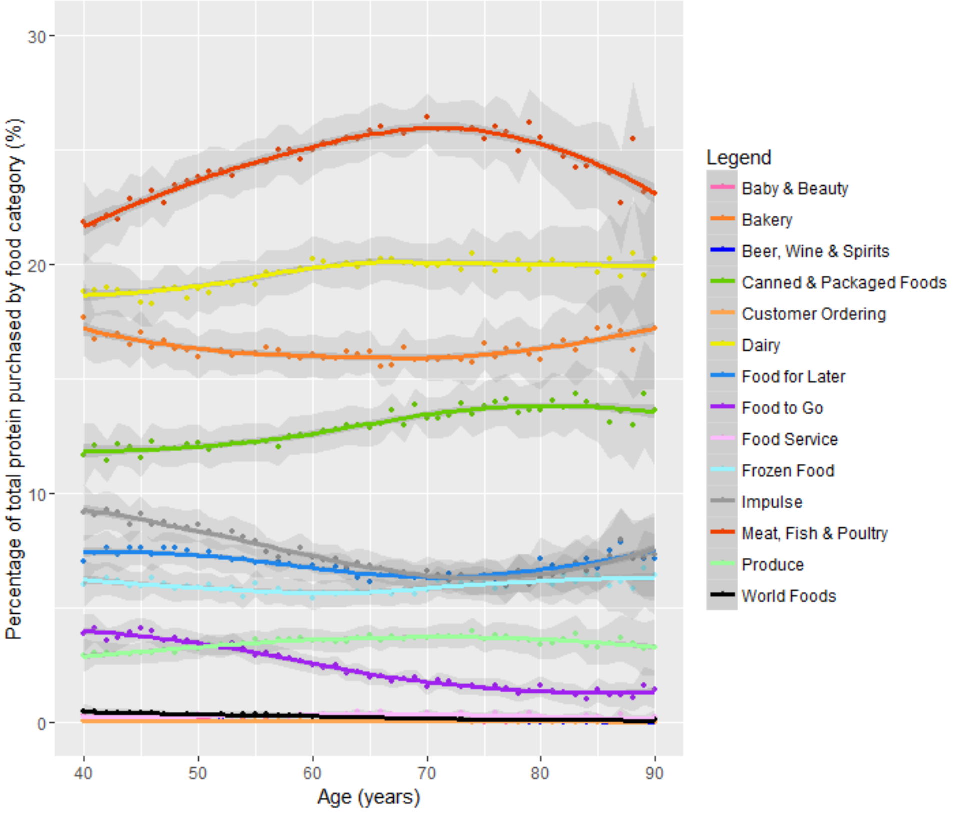

Figure F2: Percentage of protein purchased across food group by age for females in index of multiple deprivation quintile 2.

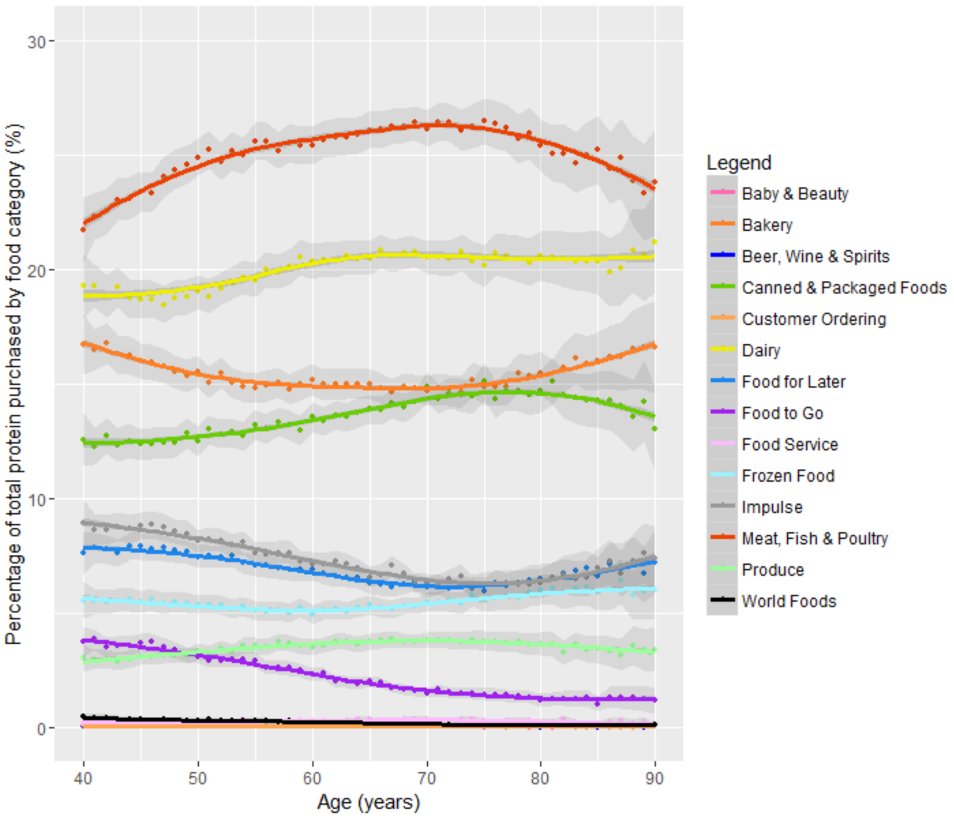

Figure F3: Percentage of protein purchased across food group by age for females in index of multiple deprivation quintile 3.

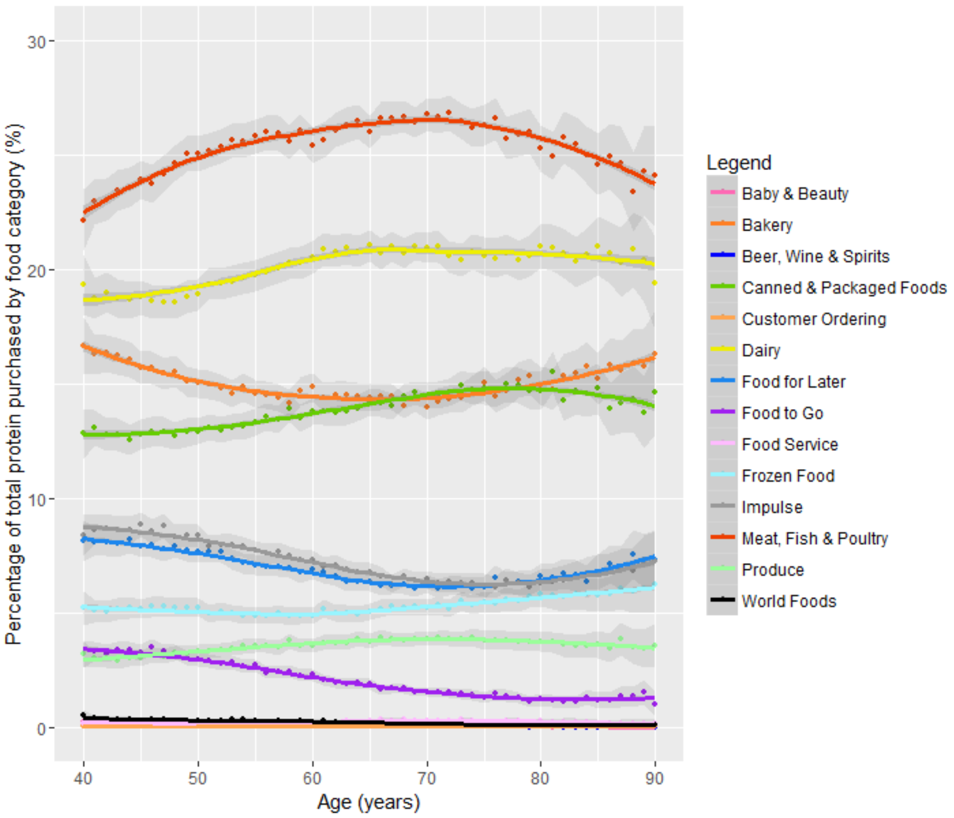

Figure F4: Percentage of protein purchased across food group by age for females in index of multiple deprivation quintile 4 (Data: High Street Supermarket, 2016-17).

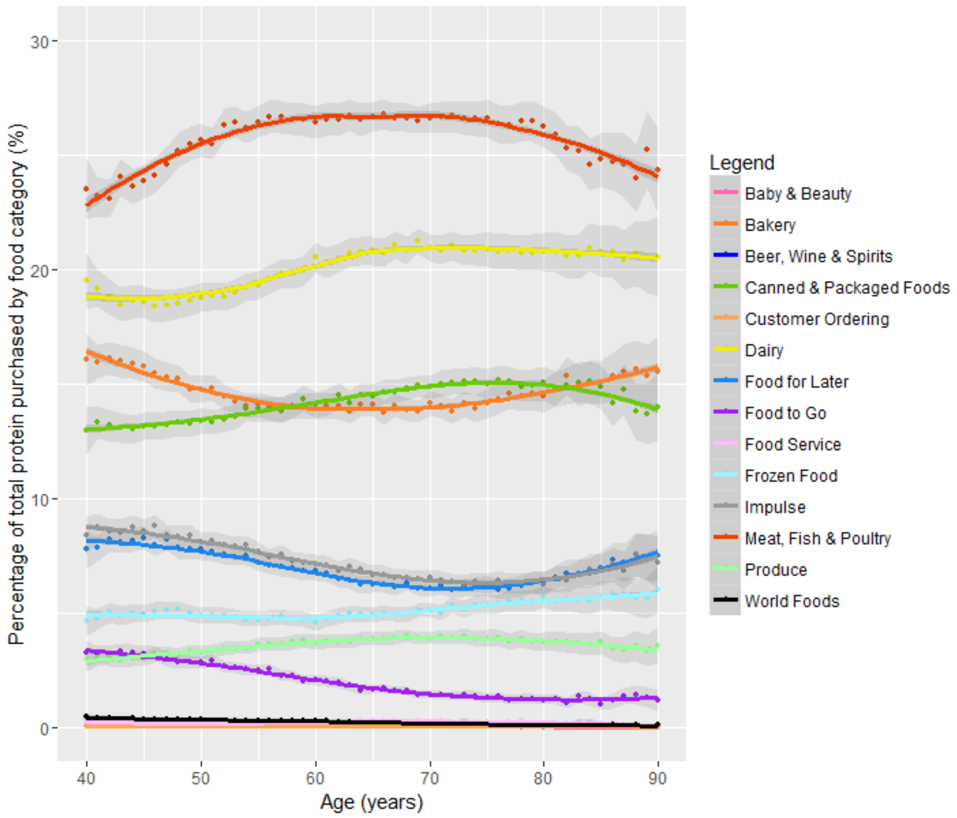

Figure F5: Percentage of protein purchased across food group by age for females in index of multiple deprivation quintile 5 (least deprived quintile) (Data: High Street Supermarket, 2016-17).
